# Temporal Associations of Alcohol and Tobacco Consumption With Cancer Mortality

**DOI:** 10.1001/jamanetworkopen.2018.0713

**Published:** 2018-07-13

**Authors:** Heng Jiang, Michael Livingston, Robin Room, Richard Chenhall, Dallas R. English

**Affiliations:** 1 Centre for Alcohol Policy Research, School of Psychology and Public Health, La Trobe University, Melbourne, Victoria, Australia; 2 Centre for Health Equity, Melbourne School of Population and Global Health, The University of Melbourne, Melbourne, Victoria, Australia; 3 Centre for Social Research on Alcohol and Drugs, Stockholm University, Stockholm, Sweden; 4 Centre for Epidemiology and Biostatistics, Melbourne School of Population and Global Health, The University of Melbourne, Melbourne, Victoria, Australia; 5 Cancer Epidemiology Centre, Cancer Council Victoria, Melbourne, Victoria, Australia

## Abstract

**Importance:**

Understanding whether the population-level consumption of alcohol and tobacco is associated with cancer mortality is a crucial question for public health policy that has not been answered by previous studies.

**Objective:**

To examine temporal associations of alcohol and tobacco consumption with overall cancer mortality in the Australian population, looking across different sex and age groups.

**Design, Setting, and Participants:**

This population-based cohort study conducted a time series analysis (autoregressive integrated moving average models) using aggregate-level annual time series data from multiple sources. Data on alcohol consumption and tobacco consumption per capita between 1935 and 2014 among the Australian population aged 15 years and older were collected from the Australian Bureau of Statistics and Cancer Council Victoria. Analysis was conducted from June 1, 2017, to October 30, 2017.

**Exposures:**

Sex- and age-specific cancer mortality rates from 1968 to 2014 were collected from the Australian Institute Health and Welfare.

**Main Outcomes and Measures:**

Population-level cancer mortality in different sex and age groups in Australia, controlling for the effects of health expenditure.

**Results:**

Among the Australian total population aged 15 years and older in this study, 50.5% were women. Cancer death rates per 100 000 persons increased from 199 in 1968 to 214 in 1989 and then decreased steadily to 162 in 2014. Taking into account lagged effects, 1-L decreases in alcohol consumption per capita were associated with a decline of 3.9% in overall cancer mortality over a 20-year period, and 1-kg decreases in tobacco consumption per capita were associated with a 16% reduction. Alcohol consumption per capita was significantly associated with overall cancer mortality among men aged 50 to 69 years and women aged 50 years and older. Tobacco consumption per capita was found to be significantly associated with overall cancer mortality only among men aged 50 years and older.

**Conclusions and Relevance:**

In this study, alcohol consumption per capita was positively associated with overall cancer mortality among older men and women, and tobacco consumption per capita was positively associated with overall cancer mortality among older men over a 20-year period. This study provides evidence that a decrease in population-level drinking and tobacco smoking could lead to a reduction in cancer mortality.

## Key Points

**Question:**

Are changes in population-level alcohol and tobacco consumption associated with changes in overall cancer mortality?

**Findings:**

In this population-based cohort study, temporal associations of alcohol and tobacco consumption with cancer mortality overall were found using Australian time series data (1935-2014). An estimated 1-L decrease in alcohol consumption per capita and a 1-kg decrease in tobacco consumption per capita were associated with a decline of 3.9% and 16%, respectively, in overall cancer mortality across a 20-year period.

**Meaning:**

Health policy interventions that can decrease population alcohol and tobacco consumption may lead to a reduction in cancer mortality over a 20-year period.

## Teaser

This population-based cohort study examines temporal associations of alcohol and tobacco consumption with overall cancer mortality in the Australian population, looking across different sex and age groups.

## Introduction

Two systematic reviews of scientific literature on alcohol, tobacco, and cancer diseases published by the World Cancer Research Fund in 2007^1^ and the International Agency for Research on Cancer in 2012^2^ concluded that long-term alcohol and tobacco consumption increases risk of cancer of the lips, oral cavity, pharynx, larynx, lung, stomach, colorectum, breast, pancreas, and liver. There is significant epidemiological evidence linking cancer onset to alcohol consumption and tobacco smoking and confirming a strong dose-response and risk association, with a higher amount of alcohol and tobacco consumption in the long-term associated with a greater risk of cancer.^3,4,5,6,7^ However, understanding whether the population-level consumption of alcohol and tobacco is associated with cancer mortality is a crucial question for public health policy that is not answered by case-control, prospective, or retrospective studies of particular samples. There is evidence for other conditions that changing alcohol and/or tobacco consumption per capita can affect population mortality rates of liver cirrhosis and ischemic heart disease,^8,9,10^ but there has been little research at the population level focusing on cancer. We addressed whether a decrease in population-level alcohol and tobacco consumption is associated with a lower cancer mortality rate in a certain period.

Case-control and prospective studies have found that associations of alcohol consumption and tobacco smoking with cancer diseases vary across sex and age groups, which may largely be because of variations in the drinking and smoking habits among different subgroups. For example, comparing those who have ever smoked with those who have never smoked, lung cancer risk was higher among men than women in Europe, while it was the opposite in the United States.^11^ The association of cumulative cigarette smoking with lung cancer death was significantly different among different sex and age groups in Japan.^5^ Sex differences were found for esophageal and liver cancer, where the alcohol-related risk was higher in women than in men, but not for other types of cancer.^12^ We extended the analysis to also investigate whether temporal associations of population alcohol and tobacco consumption with cancer mortality differ in sex and age groups.

The association of changes in population alcohol and tobacco consumption with cancer mortality will not be fully seen in the year in which the change occurs. Previous research on liver cirrhosis estimated that approximately 20% to 30% of the effect of a change in alcohol consumption per capita on cirrhosis mortality was found within a year, with the remaining effect gradually declining over a 20-year period.^13^ A pooled analysis of drinking cessation studies suggested that past alcohol drinking was no longer associated with risk of head and neck cancer after 20 years of drinking cessation.^14^ Another pooled analysis found similar results with smoking cessation, suggesting that 20 years after quitting smoking, the risk of developing oral and pharyngeal cancers was not different from the risk for those who have never smoked.^15^ These 2 studies may imply that lagged effects of alcohol or tobacco consumption on head and neck cancer extend no longer than 20 years. A systematic review conducted by Holmes et al^16^ on the temporal effects of alcohol consumption on health consequences indicates that there have been no previous aggregate-level studies of cancer mortality to inform the specification of the appropriate lag structures. Our third research question addresses the cumulative lag effects of the population level of drinking and tobacco smoking on cancer mortalities, which are still unclear.

## Method

### Data Source

A proxy for alcohol consumption per capita was constructed using data on alcohol sales sourced from the Australian Bureau of Statistics. Data on alcohol consumption per person aged 15 years and older from 1961 to 2014 were taken from a recent synthesis of historical data,^17^ while data from earlier years (1935-1960) were extracted manually from the relevant yearbooks.^18^ Data on tobacco consumption per capita 15 years and older from 1935 to 2014 were collected from Cancer Council Victoria^19^ and Klynveld Peat Marwick Goerdeler’s report, “Illicit Tobacco in Australia.”^20^ This study followed the Strengthening the Reporting of Observational Studies in Epidemiology (STROBE) reporting guideline. The study was reviewed and approved by La Trobe University, Science, Health, and Engineering College Human Ethics Sub-Committee. Available secondary and public cancer mortality data were used and no patient was involved in this study.

Age- and sex-specific cancer mortality data from 1968 to 2014 were collected from the Australian Institute of Health and Welfare,^21^ based on the *International Classification of Diseases, Tenth Revision* (codes C00-C97, D45-D46, D47.1, and D47.3-D47.5). The death rates were age standardized for men and women separately and expressed per 100 000 persons, using indirect standardization to the 2001 population age structure.

Health expenditure per capita was used as the control variable in the analysis. Health funding is central to public health planning and clinical practice; higher cancer incidences were found to be associated with lower health expenditure, and higher health expenditure to be correlated with longer cancer survival rates.^22,23^ Moreover, medical interventions to forestall or postpone deaths from various cancers have changed radically in their effectiveness in the last 80 years, and the health expenditure variable can partly function as an indicator of progress in medical science over the study period.^23,24^ Health expenditure per capita in Australia (adjusted by purchasing power parities and presented in thousands of US dollars) from 1960 to 2014 was collected from the Organization for Economic Co-operation and Development.^25^

### Statistical Analysis

Semi-log autoregressive integrated moving average (ARIMA) models were used to estimate the association of alcohol and tobacco consumption per capita with overall cancer mortality (because the slope coefficient measures the relative change in the dependent variable for a given absolute change in the value of the explanatory variable at time *t*), as the risk for chronic diseases is a convex function of alcohol and/or tobacco intake.^26^ Details of statistical model development and analysis are elaborated in the eAppendix in the Supplement.

The coefficient values of alcohol and tobacco consumption indicate the proportional change in cancer mortality rates associated with a 1-L change in weighted alcohol consumption per capita or a 1-kg change in weighted tobacco consumption per capita and are calculated as (*e*^β^ − 1) × 100. All statistical analyses were 2-sided and performed via EViews version 7.0 (HIS Global Inc) with statistical significance set at a *P* value less than .05.

### Lag Length and Lag Effects of Alcohol and Tobacco Consumption

To our knowledge, no specific lag structure or lag length has been discussed in previous aggregate analyses on alcohol, tobacco, and cancer mortalities.^16^ In accordance with previous studies on alcohol as a risk factor for chronic diseases, such as liver cirrhosis,^8,13^ lag structures were applied in the time series models in this study to account for not only present but also past drinking and smoking as potentially affecting the cancer mortality risk. Alternative truncations after a 15-year lag and after a 20-year lag were tested. Furthermore, a composite consumption measure was used, which is a weighted sum of past and present observations based on the selected lag length.

A geometrical lag scheme was used in the estimation with λ = 0.7, as advocated by Norström and Skog.^27^ This approach builds in the lagged effects of alcohol or tobacco consumption, with higher weights placed on more recent years (eFigure 1 in the Supplement). Furthermore, the lag structure detailed by Skog^28^ was also used to build lagged alcohol or tobacco consumption on overall cancer mortality (eFigure 2 in the Supplement).

Given the uncertainty about the correct lag structure, we also conducted cross-correlation tests to explore cross-correlation relationships between alcohol and tobacco consumption per capita and rates of overall cancer mortality in Australia empirically, with a proposed 30-year lag. Cross-correlation tests were widely used to capture the lag length and lagged effects of long-term alcohol consumption.^28,29^ The results of a cross-correlation test (based on the first-differenced data with trends and autocorrelation removed prewhitening) between alcohol and tobacco consumption and overall cancer mortality are presented in Table 1, showing that the highest lagged effects were at the 14th year, and the results suggest that a 20-year lag on alcohol consumption could be applied in the cancer estimating models. The cross-correlation analyses also indicate that lag length for the associations of tobacco smoking with overall cancer mortality is 20 years, with the highest lagged effects at the 12th year. The optimum lagged length of health expenditure on cancer mortality was identified as 5 years.

**Table 1.** Cross-Correlations Between First-Differenced Overall Cancer Mortality, Alcohol, and Tobacco Consumption Per Capita and Health Expenditure Per Capita

*t*	D(Cancer)^a^					
	D(Alcohol [−*t*])^b^	Lag Effects	D(Tobacco [−*t*])^b^	Lag Effects	D(Health Expenditure [−*t*])^b^	Lag Effects
0	. |* .	0.1434	. |** .	0.1986	***| .	−0.3383
1	. | .	0.0334	. *| .	−0.0887	****| .	−0.3698
2	. *| .	−0.1241	. |* .	0.0836	.**| .	−0.2276
3	. |* .	0.0671	. | .	−0.0359	***| .	−0.3223
4	. |* .	0.0833	. |* .	0.0505	***| .	−0.2680
5	. |* .	0.0493	. | .	−0.0199	****| .	−0.3629
6	. |* .	0.1543	. *| .	−0.0621	.**| .	−0.2087
7	. |***	0.2740	. |**.	0.2194	.**| .	−0.2432
8	. |**.	0.2497	. | .	0.0213	.**| .	−0.1721
9	. |* .	0.0864	. | .	−0.0220	.**| .	−0.1922
10	. |**.	0.1733	. |**.	0.1603	.**| .	−0.1776
11	. |***	0.2855	. | .	−0.0305	.**| .	−0.1751
12	. |**.	0.2178	. |***	0.2736	. *| .	−0.1135
13	. |**.	0.2178	. |* .	0.1121	. | .	−0.0492
14	. |***	0.2986	. |* .	0.1378	. | .	−0.0370
15	. |***	0.2858	. |* .	0.0729	. | .	−0.0284
16	. |* .	0.0919	. |* .	0.1483	. | .	0.0137
17	. | .	0.0246	. | .	−0.0142	. |* .	0.0641
18	. |* .	0.1480	. |**.	0.1560	. | .	0.0289
19	. |***	0.2786	. |**.	0.1804	. |* .	0.1181
20	. |***	0.2701	. |***	0.2706	. | .	0.0164
21	. |* .	0.1432	. |* .	0.1309	. | .	0.0289
22	. |* .	0.1439	. | .	0.0225	. | .	0.0397
23	. | .	0.0225	. | .	−0.0193	. | .	0.0100
24	. | .	0.0123	. | .	−0.0002	. |* .	0.1344
25	. | .	−0.0491	. | .	−0.0182	. |* .	0.1556
26	. | .	−0.0137	. | .	−0.0037	. | .	0.0116
27	. | .	−0.0003	. | .	0.0035	. | .	0.0003
28	. | .	−0.0168	. | .	0.0019	. | .	0.0120
29	. *| .	−0.0942	. | .	−0.0187	. | .	0.0261
30	. *| .	−0.0886	. | .	0.0138	. | .	0.0200

^a^ D(cancer), D(alcohol), D(tobacco), and D(health expenditure) means using first-differenced data in the analysis. The critical values of the cross-correlation test were calculated based on ±1.96/√*n* = 0.269. The greatest lag effects of changes in alcohol and tobacco consumption on overall cancer mortality were estimated at the 14th year and 12th year, respectively. The optimum lag lengths of alcohol consumption, tobacco smoking, and health expenditure on cancer mortality were identified as 20 years, 20 years, and 5 years, respectively.

^b^ Data are shown as the cross-correlation patterns of the lag effects. “|” is zero, “*” is the lag effect, and “.” indicates the critical levels indicated in footnote a.

The estimated cross-correlation lag effects were then used to build lagged alcohol and tobacco consumption series to measure the association of alcohol and tobacco consumption with overall cancer mortalities while controlling for the preventive effects from increase in health expenditure. Three different lagged consumption models, including geometric lag-weighted, Skog lag-weighted, and cross-correlation lag-weighted consumption models, were developed to be compared, based on the model fits (*R*^2^ and confidence intervals of regression) to identify the best model for the final estimation.

## Results

### Trend of Alcohol and Tobacco Consumption and Cancer Mortality

**Figure 1.**
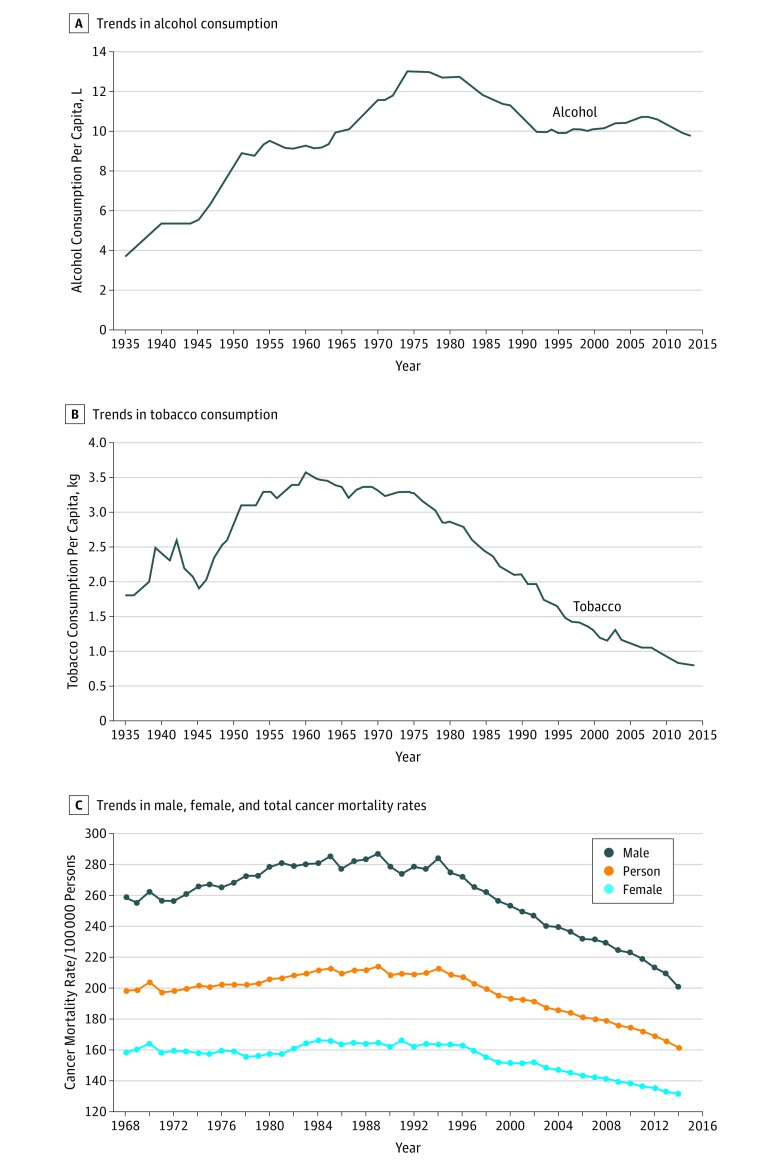
Trends in Alcohol and Tobacco Consumption and Male, Female, and Total Cancer Mortality Rates in Australia A, Alcohol consumption (in liters) per capita among persons aged 15 years and older between 1935 and 2014. B, Tobacco consumption (in kilograms) per capita among persons aged 15 years and older between 1935 and 2014. C, Male, female, and total (person) cancer mortality rates per 100 000 persons aged 15 years and older between 1968 and 2014.

**Figure 2.**
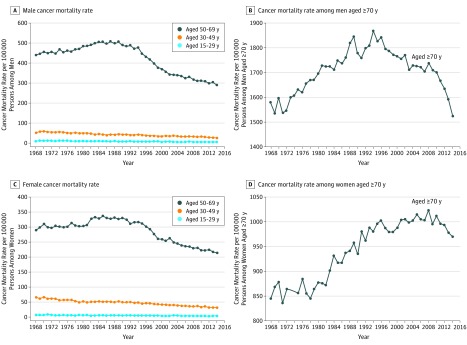
Trends of Age-Specific Cancer Mortality Rate Among Men and Women in Australia Between 1968 and 2014 A, Cancer mortality rate per 100 000 persons among men aged 15 to 29, 30 to 49, and 50 to 69 years in Australia between 1968 and 2014. B, Cancer mortality rate per 100 000 persons among men 70 years and older in Australia between 1968 and 2014. C, Cancer mortality rate per 100 000 persons among women aged 15 to 29, 30 to 49, and 50 to 69 years in Australia between 1968 and 2014. D, Cancer mortality rate per 100 000 persons among women 70 years and older in Australia between 1968 and 2014.

Among persons aged 15 years and older in Australia, 50.5% were women. Age-standardized cancer death rates per 100 000 persons in Australia increased from 199 in 1968 to 214 in 1989 and then decreased steadily to 162 in 2014 (Figure 1). Taking into account lagged effects, 1-L decreases in alcohol consumption per capita were associated with a decline of 3.9% in overall cancer mortality over a 20-year period, and 1-kg decreases in tobacco consumption per capita were associated with a 16% reduction. Alcohol consumption per capita among the Australian total population aged 15 years and older increased from 4 L to 13 L between 1935 and 1973 and decreased to 10 L by 1992, staying at the same level to 2014. Tobacco consumption per capita increased from 1.8 kg in 1935 to peak at 3.5 kg in 1960 and then decreased steadily to 0.8 kg in 2014. Mortality rates of overall cancer among persons aged 70 years and older and aged 50 to 69 years were higher than in younger age groups (Figure 2). Male and female cancer mortality rates decreased after the 1980s among people aged 50 years and older. The cancer mortality rates among the youngest male and female age groups (ages 0-29 years) were very small, and these age groups were thus excluded from further analysis.

Trends in different types of lag-weighted alcohol and tobacco consumption series and in the overall cancer mortality rate between 1968 and 2014 are presented in eFigure 3, eFigure 4, and eTable 1 in the Supplement, suggesting that cross-correlation weighted alcohol and tobacco consumption have similar trends to the trends in cancer mortality.

### Temporal Associations of Alcohol and Tobacco Consumption With Cancer Mortality

Three lagged alcohol and tobacco consumption models were constructed, based on 20-year geometric lagged weights, 20-year Skog lagged weights, and 20-year cross-correlation lagged weights. Comparing the 3 model outputs, the model with 20-year cross-correlation lagged alcohol and tobacco consumption shows significant associations of alcohol and tobacco consumption with overall, male, and female cancer mortality rates, while no significant associations were found in the geometric lagged alcohol consumption model (Table 2). The results of the Skog model suggest only a significant positive association of alcohol consumption with male cancer mortality; no association was found between tobacco consumption and cancer mortality. Furthermore, 2 models using a 15-year geometric lag and the Skog lagged model were tested in our analysis, with similar results to the 20-year models (eTable 2 in the Supplement). The cross-correlation lagged consumption model achieved a higher *R*^2^ and narrower confidence intervals in the regression analysis than the geometric and Skog lagged consumption models. Thus, we chose the 20-year cross-correlation lagged alcohol and tobacco consumption model to conduct age- and sex-specific estimations.

**Table 2.** Estimates of Temporal Associations of Alcohol and Tobacco Consumption With Overall, Male, and Female Cancer Mortality Based on 3 Different Lag Models

Lag Model	Cancer, Coefficient (95% CI)		
	Male	Female	Total
Model with 20-y geometric lags			
Alcohol	0.005 (−0.036 to 0.046)	0.031 (−0.012 to 0.074)	0.014 (−0.021 to 0.049)
Tobacco	−0.078 (−0.303 to 0.147)	−0.154 (−0.401 to 0.093)	−0.093 (−0.289 to 0.103)
Health expenditure (5-y geometric lag)	−0.105 (−0.195 to −0.015)^a^	−0.161 (−0.212 to −0.110)^a^	−0.124 (−0.200 to −0.048)^a^
Constant	−0.000 (−0.016 to 0.016)	−0.001 (−0.015 to 0.013)	−0.001 (−0.015 to 0.013)
Model specification	0,1,0	0,1,0	0,1,0
Box-Ljung *Q* (lag 10)^b^	7.787	8.388	6.147
*P* value	.65	.59	.80
*R*^2^	0.155	0.318	0.260
Model with 20-y Skog lags			
Alcohol	0.061 (0.002 to 0.120)^a^	0.018 (−0.037 to 0.073)	0.038 (−0.007 to 0.083)
Tobacco	−0.239 (−0.502 to 0.024)	−0.114 (−0.363 to 0.135)	−0.170 (−0.368 to 0.028)
Health expenditure (5-y Skog lag)	−0.158 (−0.254 to −0.062)^c^	−0.105 (−0.195 to −0.015)^a^	−0.128 (−0.201 to −0.055)^c^
Constant	−0.005 (−0.023 to 0.013)	−0.002 (−0.018 to 0.014)	−0.003 (−0.017 to 0.011)
Model specification	0,1,0	0,1,0	0,1,0
Box-Ljung *Q* (lag 10)^b^	10.054	7.362	6.999
*P* value	.44	.69	.73
*R*^2^	0.36	0.16	0.35
Model with 20-y cross-correlation lags			
Alcohol	0.043 (0.012 to 0.074)^a^	0.035 (0.010 to 0.060)^a^	0.038 (0.014 to 0.062)^c^
Tobacco	0.266 (0.115 to 0.417)^c^	0.083 (−0.066 to 0.232)	0.151 (0.078 to 0.224)^d^
Health expenditure (5-y cross-correlation lag)	−0.046 (−0.148 to 0.056)	−0.042 (−0.151 to 0.068)	−0.047 (−0.127 to 0.033)
Constant	0.010 (0.000 to 0.020)^a^	−0.002 (−0.012 to 0.008)^a^	0.005 (−0.003 to 0.013)
Model specification	1,1,0	0,1,1	1,1,1
Box-Ljung *Q* (lag 10)^b^	9.546	4.996	11.658
*P* value	.39	.84	.17
*R*^2^	0.582	0.467	0.589

^a^ *P* < .05.

^b^ The Box-Ljung *Q* test is a diagnostic tool used to test the lack of fit of a time series model, and a *P* value of the Box-Ljung *Q* test greater than .10 indicates the test rejects the null hypothesis of lack of fit of the time series model.

^c^ *P* < .01.

^d^ *P* < .001.

The detailed age- and sex-specific model outputs of the cross-correlation lagged alcohol and tobacco consumption models are summarized in Table 3. The results suggest that a 1-L decrease in alcohol consumption per capita was associated with a male cancer mortality reduction of 4.4% [(*e*^0.043^ − 1) × 100] and a female cancer mortality reduction of 3.6% [(*e*^0.035^ − 1) × 100] across a 20-year period, while a 1-kg decrease in tobacco consumption per capita was associated with a male cancer mortality reduction of 30% across a 20-year period, controlling for the trend of health expenditure per capita. The female cancer mortality was not significantly associated with tobacco smoking. Compared with other age groups, alcohol consumption per capita was significantly associated with mortality among men aged 50 to 69 years and women 50 years and older. Tobacco consumption per capita with cancer mortality among men aged 50 years and older only. The results of model validation tests (Box-Ljung *Q*) suggest that all estimated models were satisfactory with regard to autocorrelation of residuals, and there were no nonnormal errors in the residuals with *P* values greater than .10 (Table 2 and Table 3).

**Table 3.** Estimates of Temporal Associations of Alcohol and Tobacco Consumption With Sex- and Age-Specific Cancer Mortality Based on the Cross-Correlation Lag Model

Characteristic	Consumption, Coefficient (95% CI)		Model Specification	Box-Ljung *Q* (Lag 10)	
	Alcohol	Tobacco		*Q* Statistic	*P* Value^a^
Male by age, y					
30-49	0.032 (−0.137 to 0.201)	0.137 (−1.080 to 1.354)	1,1,0	6.404	.70
50-69	0.095(0.040 to 0.150)^b^	0.170 (0.029 to 0.311)^c^	1,1,0	10.883	.28
≥70	0.016 (−0.035 to 0.067)	0.263 (0.075 to 0.451)^d^	0,1,0	8.350	.60
Subtotal	0.043 (0.012 to 0.074)^c^	0.266 (0.115 to 0.417)^d^	1,1,0	9.546	.39
Female by age, y					
30-49	0.022 (−0.051 to 0.095)	0.070 (−0.181 to 0.321)	1,1,0	5.080	.75
50-69	0.059 (0.008 to 0.110)^d^	0.063 (−0.151 to 0.277)	0,1,0	8.576	.57
≥70	0.042 (0.020 to 0.064)^c^	0.067 (−0.015 to 0.149)	0,1,1	7.357	.60
Subtotal	0.035 (0.010 to 0.060)^c^	0.083 (−0.066 to 0.232)	0,1,1	4.996	.84

^a^ The Box-Ljung *Q* test is a diagnostic tool used to test the lack of fit of a time series model, and a *P* value of the Box-Ljung *Q* test greater than .10 indicates the test rejects the null hypothesis of lack of fit of the time series model.

^b^ *P* < .001.

^c^ *P* < .05.

^d^ *P* < .01.

## Discussion

This population-based cohort study provides the first evidence, to our knowledge, from an aggregate-level temporal analysis that a decrease in population-level drinking and smoking can reduce overall cancer mortality, particularly among older men and women. The results suggest that decreases of 1 L in pure alcohol consumption per capita were associated with a 3.9% reduction in overall cancer mortality across a 20-year period, while decreases of 1 kg per capita in tobacco consumption were associated with a 16% decrease in overall cancer mortality across a 20-year period.

The consistent results between our aggregate analyses and Australian epidemiological studies^30^ and burden-of-disease studies on alcohol and cancer^31^ suggest that reducing alcohol consumption at the population level could have greater preventive effects on cancer mortality for men than for women. Furthermore, our results suggest that 12.8% of total cancer mortality was attributable to tobacco smoking. This is consistent with the results of a recent Australian tobacco epidemiological study^32^ that found that 13% of cancer deaths in Australian were attributable to tobacco smoke. Our findings also suggest that if the current Australian population drinking level (9 L per capita in 2014) could be reduced to 6 L per capita, the overall cancer mortality rate would be reduced by 11.7% across a 20-year period. Our estimate of the association of alcohol consumption with cancer mortality is greater than the findings from current epidemiological studies that 6% of cancer mortality was attributable to alcohol use in 2012.^33^ However, it is still unknown whether there is a threshold below which further reductions in population drinking do not significantly further reduce cancer deaths. More analyses on particular types of cancers (eg, oral cavity, larynx, and liver) related to alcohol use could help to explain this inconsistency.

The associations of alcohol and tobacco consumption per capita with cancer mortality were found only among older groups. This can be seen as reflecting the long-term effects of alcohol and tobacco consumption on the development of malignant neoplasms in the human body, although, as noted, more work is needed to specify the appropriate lag structures to reflect the time lag between changes in consumption, incidence of specific types of cancer, and mortality. Stronger associations between alcohol consumption and cancer mortality were found particularly among those aged 50 to 69 years. With people aged 70 years and older, competing causes of death such as accidental falls, cardiovascular diseases, and infectious diseases may reduce the toll from cancer. In contrast, an association between tobacco consumption per capita and cancer mortality was only found among men in our analysis. The possible reason is that the prevalence of smoking among women was much lower than among men before the 1980s. In the 1940s, the prevalences were 26% for women and 72% for men; by the 1980s, they were 30% for women and 41% for men.^19^ Other factors may also have influenced the sex-specific findings, such as greater access to health care services or greater general health and well-being among women in Australia.^34^

Our study also provides an answer to the question often raised by public health researchers and health professionals—what are the lag structures and cumulative temporal effects of alcohol drinking and tobacco smoking on cancer mortality? The results of lag length and lag effects analyses suggested that there were 20 years of cumulative lagged effects of drinking and smoking on cancer mortality at the population level, with the highest lagged effects in the 14th and 12th years, respectively. The proposed lag effects estimation and time series analyses served as an experiment in the use of the time series method, which can be used to analyze how the changes in alcohol and tobacco consumption could affect cancer deaths in the long-term.

Previous studies found that higher health expenditures were associated with increased cancer incidence and decreased cancer mortality.^35^ We also found significant and negative associations between health expenditure and cancer mortality in the age-specific estimations, particularly in older groups (eTable 3 in the Supplement), suggesting that an increase in health expenditure per person can reduce cancer mortality rates or increase cancer survivor rates within a 5-year period. However, there was no association between health expenditure per capita and cancer mortality in the overall model. The low cancer mortality and incidence rates in younger groups could affect the overall link between health expenditure and cancer diseases. Additionally, an improvement in cancer survival in more recent years was expected owing to the new drugs and technology being used and better diagnosis and treatment in the early stage. This may also affect the temporal associations of drinking and smoking with cancer mortality in Australia.

The synergistic effects of tobacco smoking and alcohol consumption on cancer mortality were discussed in previous studies,^36,37^ and a greater joint effect on cancers was found for combined consumption of alcohol and tobacco than for consuming either of them alone. Research evidence has also found heavy drinking appears to be a risk factor for heavy smoking and vice versa, suggesting that any control policy with respect to one behavior should take into account the potential involvement of the other.^38^ While the synergistic associations cannot be tested in this time series analysis, the effects of drinking and smoking with cancer mortality were both examined concurrently in our model while controlling for the effects of health expenditure. Public health advocates and policy makers on alcohol and tobacco are urged to work together and learn from each other to minimize the long-term adverse health effects on cancer from these 2 risky behaviors.

From a health policy perspective, alcohol and tobacco control interventions have led to reduced population-level alcohol and tobacco consumption in the last 5 decades.^19,39^ Some key examples include the release of the Royal College of Physicians and US Surgeon General reports on smoking in relation to cancer and other diseases from 1962 to 1964,^40^ public campaigns about the dangers of tobacco in 1960s, banning cigarette advertisements on television and radio in 1976,^41^ introducing random breath testing programs to counter drunk driving since 1976, and setting up national drinking guidelines in 1986.^42^ Indexed excise regimes for both tobacco and alcohol in Australia have also ensured relatively high prices for many products (although not all types of alcohol).^43^ It is expected that these key policy interventions may have generated substantial preventive effects on cancer deaths in Australia. From a clinical perspective, clinical interventions that help to reduce alcohol consumption and tobacco smoking (eg, brief cessation counseling and the provision of medication to help smokers quit as well as brief interventions for heavy drinking) are likely to contribute to reduced risk of cancer among patients.

### Limitations

Our study had limitations. We did not take into account other confounding factors associated with cancer mortality, such as levels of physical activity and sugar consumption, air pollution status, and some key policy interventions on alcohol or tobacco consumption in the study period. However, the use of first-differenced data and of controls for serial correlation can partially control for trends in unmeasured confounders, and factors that cannot be measured in our models are also likely to be captured by the model residuals. The long study period is an advantage for model estimation, making possible the more conservative specifications used and estimations of 30-year lag effects on alcohol, tobacco, and cancer mortality. Because of the paucity of available data, the prevalence rates of drinking and smoking were not considered in the model; trends in sex- and age-specific alcohol and tobacco consumption in the longer term are unavailable in Australia. Thus, caution is suggested in the interpretation of age- and sex-specific findings. There has been marked improvement in treatment of cancer outcomes, which may diminish the likelihood of cancer mortality (ie, people are not dying but living longer with cancer or going into remission). This effect was partially captured by the health expenditure variable used as a potential confounder in our model.^24^ Furthermore, unrecorded alcohol and tobacco smoking is not considered in this study, although it is unlikely to have a substantial impact on our estimations, as unrecorded alcohol and tobacco consumption are less than 5% of the total consumption (eg, Australian unrecorded alcohol consumption was 0.1 L per capita in 2005).^44^

## Conclusions

The positive temporal associations of both alcohol and tobacco consumption with cancer mortality found in this study provide confirmatory evidence that public health policies of reducing population drinking and smoking can reduce cancer mortality over a 20-year period in Australia. Future research on associations between alcohol and tobacco consumption and specific types of cancer mortalities is needed. In a more global perspective, future studies using multicountry cancer mortality data from the World Health Organization cancer database could help to better understand the associations in different socioeconomic status and health policy environments and identify effective policy or clinical interventions to reduce alcohol- and tobacco-related cancer diseases.

## Notes

Supplement. **eAppendix.** Statistical Model Development and Analysis **eFigure 1.** Geometric Distribution of Lag Effects of Alcohol or Tobacco Consumption on Cancer Mortality in the Past 15 and 20 Years **eFigure 2.** Skog’s Lag Distribution of Lag Effects of Alcohol or Tobacco Consumption on Cancer Mortality in the Past 15 and 20 Years **eFigure 3.** Trends in Alcohol Consumption and Lagged Alcohol Consumption Series and Overall Cancer Mortality Rate per 100 000 Population **eFigure 4.** Trends in Tobacco Consumption and Lag Weighted Tobacco Consumption and Overall Cancer Mortality Rate per 100 000 Population **eTable 1.** Unit Root Test for Stationarity of Time Series **eTable 2.** Estimates of 15 Years Geometric and Skog’s Lagged Effects of Alcohol and Tobacco Consumption, and Overall Cancer Mortality **eTable 3.** Temporal Associations Between Alcohol and Tobacco Consumption and Gender- and Age-Specific Cancer Mortality Based on the Cross-Correlation Lag Model **eReferences**
