# Supplementary material for: Temporal Associations of Alcohol and Tobacco Consumption With Cancer Mortality
Source: JAMA Netw Open. 2018 Jul 13;1(3):e180713. doi: 10.1001/jamanetworkopen.2018.0713 (PMC6324312; doi:10.1001/jamanetworkopen.2018.0713)
Supplement: Supplement. — eAppendix. Statistical Model Development and Analysis eFigure 1. Geometric Distribution of Lag Effects of Alcohol or Tobacco Consumption on Cancer Mortality in the Past 15 and 20 Years eFigure 2. Skog’s Lag Distribution of Lag Effects of Alcohol or Tobacco Consumption on Cancer Mortality in the Past 15 and 20 Years eFigure 3. Trends in Alcohol Consumption and Lagged Alcohol Consumption Series and Overall Cancer Mortality Rate per 100 000 Population eFigure 4. Trends in Tobacco Consumption and Lag Weighted Tobacco Consumption and Overall Cancer Mortality Rate per 100 000 Population eTable 1. Unit Root Test for Stationarity of Time Series eTable 2. Estimates of 15 Years Geometric and Skog’s Lagged Effects of Alcohol and Tobacco Consumption, and Overall Cancer Mortality eTable 3. Temporal Associations Between Alcohol and Tobacco Consumption and Gender- and Age-Specific Cancer Mortality Based on the Cross-Correlation Lag Model eReferences [file jamanetwopen-1-e180713-s001.pdf]

Supplementary Online Content

Jiang H, Livingston M, Room R, Chenhall R, English DR. Temporal associations of alcohol and tobacco consumption with cancer mortality. *JAMA Netw Open*. 2018;1(3):e180713. doi:10.1001/jamanetworkopen.2018.0713

eAppendix. Statistical Model Development and Analysis
eFigure 1. Geometric Distribution of Lag Effects of Alcohol or Tobacco Consumption on Cancer Mortality in the Past 15 and 20 Years
eFigure 2. Skog's Lag Distribution of Lag Effects of Alcohol or Tobacco Consumption on Cancer Mortality in the Past 15 and 20 Years
eFigure 3. Trends in Alcohol Consumption and Lagged Alcohol Consumption Series and Overall Cancer Mortality Rate per 100 000 Population
eFigure 4. Trends in Tobacco Consumption and Lag Weighted Tobacco Consumption and Overall Cancer Mortality Rate per 100 000 Population
eTable 1. Unit Root Test for Stationarity of Time Series
eTable 2. Estimates of 15 Years Geometric and Skog's Lagged Effects of Alcohol and Tobacco Consumption, and Overall Cancer Mortality
eTable 3. Temporal Associations Between Alcohol and Tobacco Consumption and Gender- and Age-Specific Cancer Mortality Based on the Cross-Correlation Lag Model
eReferences

This supplementary material has been provided by the authors to give readers additional information about their work.

© 2018 Jiang H et al. *JAMA Network Open*.

eAppendix. Statistical Model Development and Analysis

Data collection

A proxy for per-capita alcohol consumption was constructed, using data on alcohol sales sourced from the Australian Bureau of Statistics (ABS). Data on alcohol consumption per person aged 15+ for the years 1961 to 2014 are taken from a recent synthesis of historical data,[1] while data from earlier years (1935-1960) were extracted manually from the relevant yearbooks [2] [e.g. Commonwealth Bureau of Census and Statistics report], and converted from gallons or proof gallons to litres of pure alcohol. This was then converted to litres of pure alcohol per resident aged 15 and older, using population data provided by the Australian Institute of Health and Welfare (AIHW).[3] Data on per capita tobacco consumption (aged 15+) from 1935 to 2014 were collected from Cancer Council Victoria[4] and KPMG's report, *Illicit Tobacco in Australia*.[5]

Time series model

The autoregressive integrated moving average (ARIMA) modelling technique was employed to estimate the association between per-capita alcohol consumption and overall cancer mortality. ARIMA models require stationary time series to reduce the risk of obtaining a spurious relation between two series that have common trends.[6] The Augmented Dickey-Fuller (ADF) unit root test is commonly used for testing for stationarity.[7] Furthermore, the error term (which includes explanatory variables not considered in the model) is allowed to have a temporal structure that is modelled and estimated in terms of autoregressive or moving average parameters.[8] In most cases, a differencing of the time series is sufficient to eliminate non-stationarity.[9] In this study, a semi-log ARIMA model was selected (because the slope coefficient measures the relative change in dependent variable for a given absolute change in the value of the explanatory variable at time *t*), as the risk for chronic diseases is a convex function of alcohol or/and tobacco intake.[10] The final model can be written as follows:

$$\Delta LogCM_t = \alpha + \beta \Delta WALC_t + \gamma \Delta WTOB_t + \mu \Delta C_{i,t} + \Delta E_t$$

where Δ is the differencing operator, $LogCM_t$ is the natural logarithm of mortality rates of overall cancer diseases in Australia per 100,000 inhabitants, $WALC_t$ is lag weighted per-capita alcohol consumption, $WTOB_t$ is lag weighted per-capita tobacco consumption, $C_{i,t}$ are the other control variables considered in the estimation, i is number of control variables, μ is the coefficient values of the control variables, E_t is the error term including other causal factors, and α is the constant. The coefficient values β or γ indicate the proportional change in cancer mortality rate associated with a one-litre change in weighted per-capita alcohol consumption or a 1 kg change in weighted per capita tobacco consumption $(e^{\beta} - 1) \times 100$.

The model fit was evaluated with the aid of the Box-Ljung portmanteau test of the first 10 autocorrelations, $Q(10)$. The model structures used are reported below, alongside the output of the models. All statistical analyses were undertaken via E-views 7.0.

© 2018 Jiang H et al. *JAMA Network Open*.

Lag length and lag weight

Geometric lag weight

A geometrical lag scheme was used in the estimation with λ=0.7. This approach builds in the lagged effects of alcohol or tobacco consumption, with higher weights placed on more recent years (shown in eFigures 1).

Geometric weight = $\frac{X_n+0.7^2 X_{n-1}+\cdots+0.7^{n-1} X_2+0.7^n X_1}{1+0.7+0.7^2+\cdots+0.7^{n-1}+0.7^n}$

© 2018 Jiang H et al. *JAMA Network Open*.

eFigure 1. Geometric Distribution of Lag Effects of Alcohol or Tobacco Consumption on Cancer Mortality in the Past 15 and 20 Years

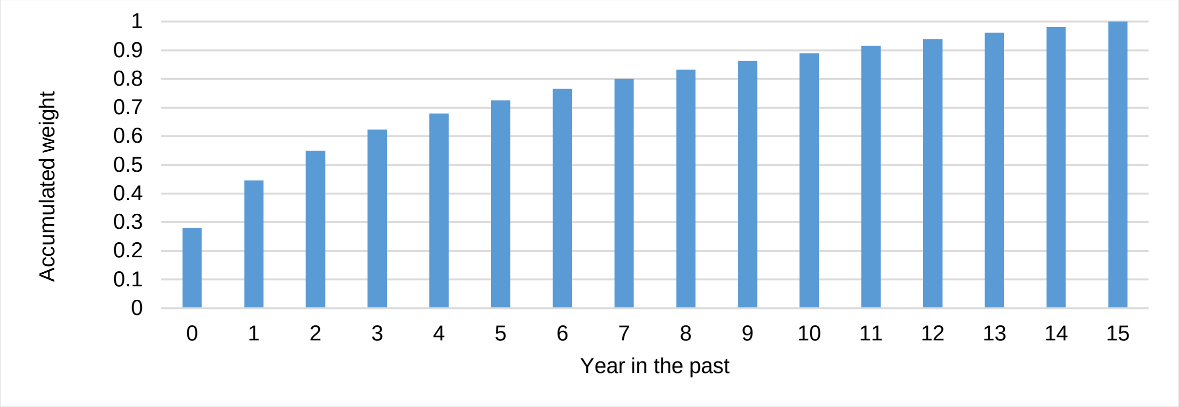

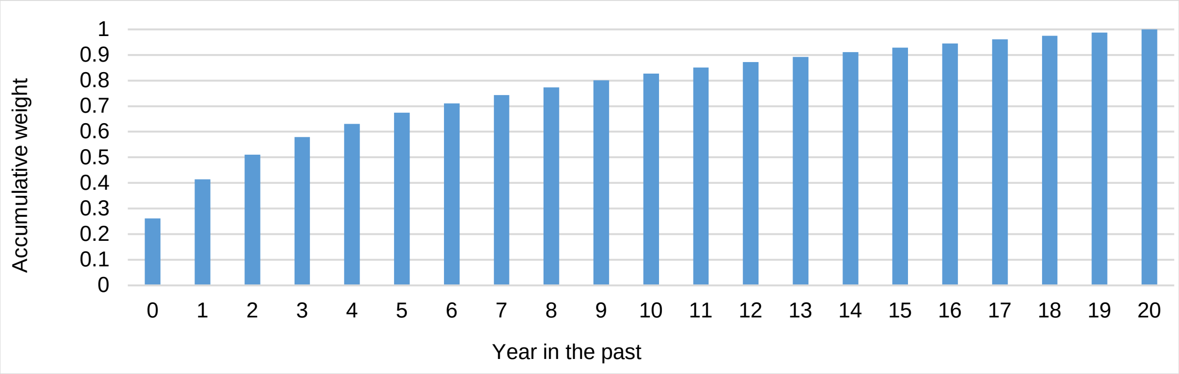

Skog's lag weight

A three parameter lag structure developed by Skog [11] was also used to build lagged alcohol or tobacco consumption on cancer mortality.

Skog lag weight = $W_t = p\theta_1^t + (1-p)\theta_2^t$

Where W_t is the weight of alcohol or tobacco consumption in year t, θ_1 is the lag parameter for the short-term impact, θ_2 for long-term impact, and p determines their relative imprtance. Based on the previous studies on alcohol consumption and liver cirrhosis mortality, p =0.80, θ_1=0.50 θ_2= 0.93 were used in our estimation (shown in eFigures 2).

© 2018 Jiang H et al. *JAMA Network Open*.

eFigure 2. Skog's Lag Distribution of Lag Effects of Alcohol or Tobacco Consumption on Cancer Mortality in the Past 15 and 20 Years

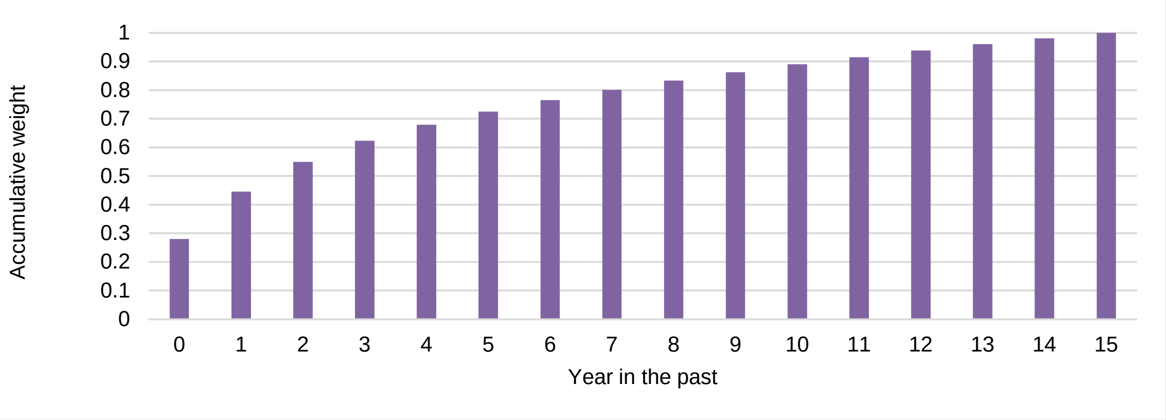

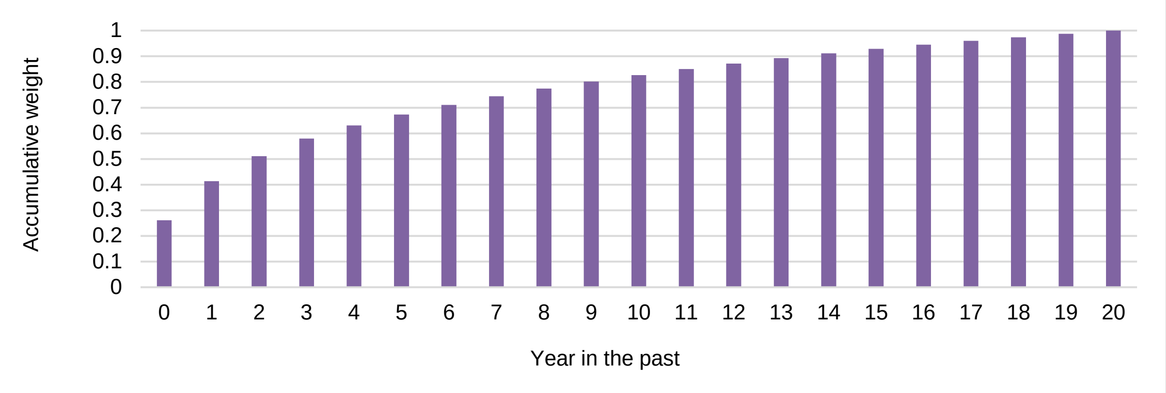

Unit root test

The ADF unit root test (eTable 1) is employed to test the stationarity of the time series in this study, suggesting that alcohol consumption, tobacco consumption, health expenditure, and all gender- and age-specific cancer mortalities are non-stationary in data at the untransformed level, and become stationary after first differencing at the significance level of 0.05.

© 2018 Jiang H et al. *JAMA Network Open*.

eFigure 3. Trends in Alcohol Consumption and Lagged Alcohol Consumption Series and Overall Cancer Mortality Rate per 100 000 Population

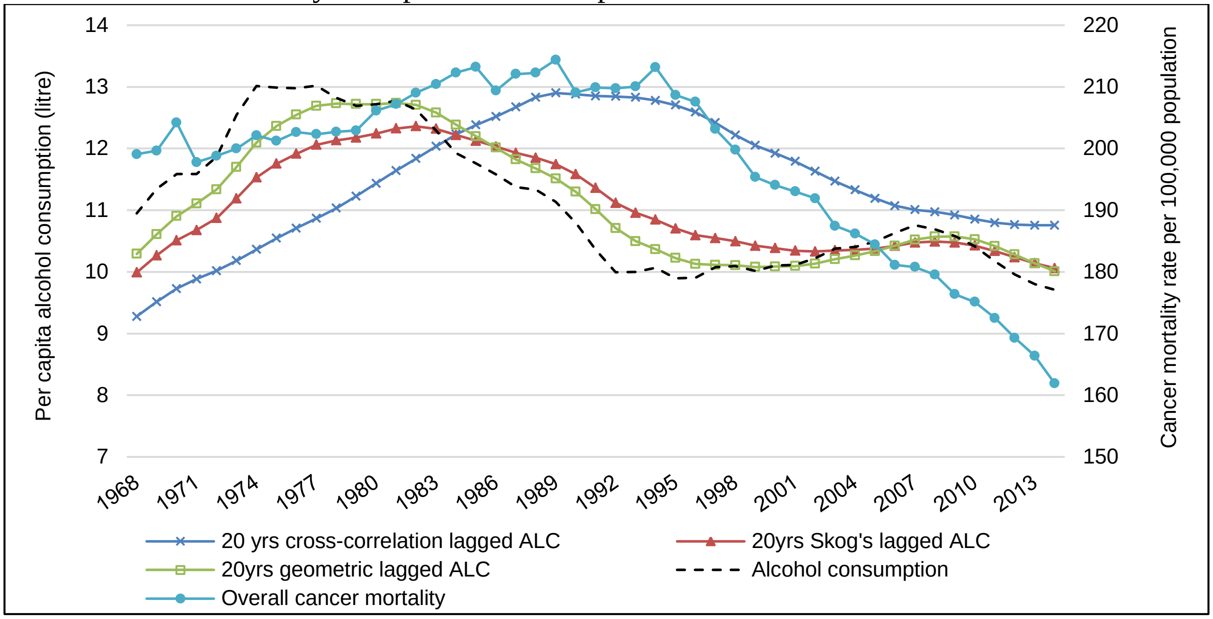

Sensitivity analysis using 15 years geometric and Skog's lagged effects of alcohol consumption

© 2018 Jiang H et al. *JAMA Network Open*.

eFigure 4. Trends in Tobacco Consumption and Lag Weighted Tobacco Consumption and Overall Cancer Mortality Rate per 100 000 Population

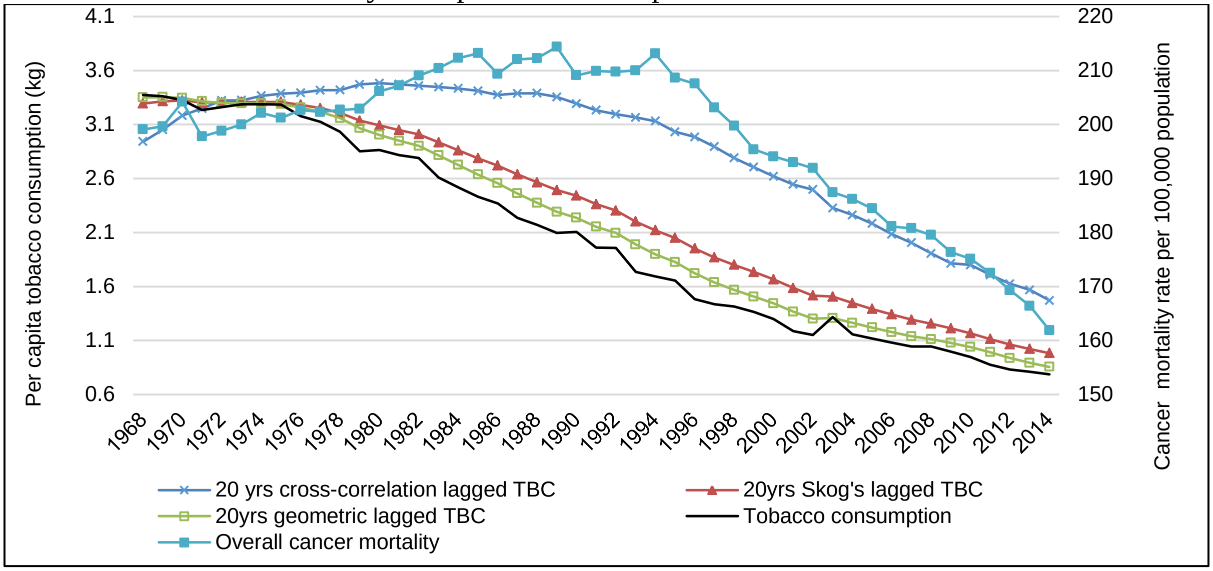

Sensitivity analysis using 15 years geometric and Skog's lagged effects of tobacco consumption

© 2018 Jiang H et al. *JAMA Network Open*.

eTable 1. Unit Root Test for Stationarity of Time Series

	Augmented Dickey-Fuller Test on level data		Augmented Dickey-Fuller Test on first differenced data	
	T-statistics	P-value	T-statistics	P-value
Alcohol consumption per capita	-1.795	0.379	-2.014	0.043
Tobacco consumption per capita	-1.976	0.599	-7.361	0.000
Health expenditure per capita	1.244	0.999	-3.594	0.040
Male cancer mortality	-0.483	0.981	-8.799	0.000
Female cancer mortality	-0.788	0.959	-7.532	0.000
Overall cancer mortality	-0.152	0.992	-8.317	0.000
Male cancer mortality 30-49 years	-0.576	0.866	-9.512	0.000
Male cancer mortality 50-69 years	-0.799	0.809	-7.585	0.000
Male cancer mortality 70 yr+	-0.868	0.790	-7.032	0.000
Female cancer mortality 30-49 years	-0.339	0.911	-10.980	0.000
Female cancer mortality 50-69 years	-1.676	0.746	-6.573	0.000
Female cancer mortality 70 yr+	-0.553	0.977	-10.155	0.000

Trends of different types of lag weighted alcohol and tobacco consumption and overall cancer mortality between 1968 and 2014

© 2018 Jiang H et al. *JAMA Network Open*.

eTable 2. Estimates of 15 Years Geometric and Skog's Lagged Effects of Alcohol and Tobacco Consumption, and Overall Cancer Mortality

	Male		Female		Total	
	Coef.	(95% CI)	Coef.	(95% CI)	Coef.	(95% CI)
Model with 15 years geometric lag weight						
Alcohol	0.027	(-0.014, 0.068)	0.000	(-0.039, 0.039)	0.013	(-0.018, 0.044)
Tobacco	-0.135	(-0.378, 0.108)	-0.053	(-0.276, 0.170)	-0.092	(-0.274, 0.090)
Health spending	-0.175***	(-0.269, -0.081)	-0.112*	(-0.198, -0.026)	-0.139***	(-0.210, -0.068)
Constant	0.001	(-0.015, 0.017)	0.002	(-0.014, 0.018)	0.002	(-0.010, 0.014)
Model specification	*0,1,0*		*0,1,0*		*0,1,0*	
Box-Ljung *Q (lag 10)*	*8.213, p=0.608*		*7.871, p=0.641*		*6. 422, p=0.779*	
R-square	*0.313*		*0.150*		*0.313*	
Model with 15 year Skog lag weight						
Alcohol	0.050*	(-0.001, 0.101)	0.014	(-0.033, 0.061)	0.031	(-0.006, 0.068)
Tobacco	-0.213	(-0.460, 0.034)	-0.105	(-0.105, -0.338)	-0.154	(-0.340, 0.032)
Health spending	-0.154**	(-0.250, -0.058)	-0.101*	(-0.101, -0.191)	-0.124	(-0.197, -0.051)
Constant	-0.005	(-0.023, 0.013)	-0.002	(-0.002, -0.018)	-0.003	(-0.017, 0.011)
Model specification	*0,1,0*		*0,1,0*		*0,1,0*	
Box-Ljung *Q (lag 10)*	*9.818, p=0.457*		*7.307, p=0.696*		*6.714, p=0.752*	
R-square	*0.353*		*0.156*		*0.345*	

Note: ***p<0.001, **p<0.01, *p<0.05; S.E. is standard errors.

© 2018 Jiang H et al. *JAMA Network Open*.

eTable 3. Temporal Associations Between Alcohol and Tobacco Consumption and Gender- and Age-Specific Cancer Mortality Based on the Cross-Correlation Lag Model

	Male cancer		Female cancer	
	Coef.	(95% CI)	Coef.	(95% CI)
30-49 years				
Alcohol	0.032	(-0.137, 0.201)	0.022	(-0.051, 0.095)
Tobacco	0.137	(-1.80, 1.354)	0.070	(-0.181, 0.321)
Health expenditure (5 years geometric lag)	-0.051	(-0.435, 0.333)	-0.104	(-0.339, 0.131)
Constant	-0.006	(-0.039, 0.027)	-0.014	(-0.034, 0.006)
Model specification	*1,1,0*		*1,1,0*	
Box-Ljung *Q (lag 10)*	6.404, *p*=0.699		5.080, *p*=0.749	
R-square	*0.120*		*0.451*	
50-69 years				
Alcohol	0.095***	(0.040, 0.150)	0.059**	(0.028, 0.090)
Tobacco	0.170*	(-0.030, 0.370)	0.063	(-0.151, 0.277)
Health expenditure (5 years Skog lag)	-0.149*	(-0.269, -0.029)	-0.105	(-0.346, 0.136)
Constant	-0.016	(-0.026, -0.006)	-0.016	(-0.037, 0.004)
Model specification	*1,1,0*		*0,1,0*	
Box-Ljung *Q (lag 10)*	10.883, *p*=0.284		8.576, *p*=0.573	
R-square	*0.637*		*0.477*	
70+ years				
Alcohol	0.016	(-0.035, 0.067)	0.042*	(0.020, 0.064)
Tobacco	0.263**	(0.0075, 0.451)	0.067	(-0.015, 0.149)
Health expenditure (5 years cross-correlation lag)	-0.174*	(-0.525, 0.177)	0.118*	(0.028, 0.208)
Constant	0.023	(-0.004, 0.050)	0.010	(-0.045, 0.065)
Model specification	*0,1,0*		*0,1,1*	
Box-Ljung *Q (lag 10)*	8.350, *p*=0.595		7,357, *p*=0.600	
R-square	0.534		*0.468*	

Note: ****p*<0.001, ***p*<0.01, **p*<0.05; S.E. is standard errors.

© 2018 Jiang H et al. *JAMA Network Open*.

eReferences

1. ABS, Apparent Consumption of Alcohol, Australia, 2013-14 (Cat: 4307.0.55.001). 2015, Australian Bureau of Statistics: Canberra.
2. Commonwealth Bureau of Census and Statistics, Official Yearbook of the Commonwealth of Australia 1911-1959. 1960, Commonwealth Bureau of Census and Statistics,: Canberra.
3. AIHW, Australian Cancer Incidence and Mortality (ACIM) books. 2016, Australian Institute of Health and Welfare: Canberra.
4. Scollo, M. and M. Winstanley, Tobacco in Australia: Facts and issues, C.C. Victoria, Editor. 2015, Cancer Council Victoria: Melbourne.
5. KPMG, Illicit tobacco in Australia 2016, London, DOI: https://home.kpmg.com/content/dam/kpmg/pdf/2016/04/australia-illict-tobacco-2015.pdf.
6. Ramstedt, M., Per capita alcohol consumption and liver cirrhosis mortality in 14 European countries. Addiction, 2001. 96: S19-S33.
7. Dickey, D.A. and W.A. Fuller, Distribution of the estimators for autoregressive time series with a unit root. Journal of the American Statistical Association, 1979. 74(366): 427-431.
8. Stickley, A., T. Jukkala, and T. Norstrom, Alcohol and suicide in Russia, 1870-1894 and 1956-2005: evidence for the continuation of a harmful drinking culture across time?(Report). Journal of Studies on Alcohol and Drugs, 2011. 72(2): 341(7).
9. Norström, T., Per capita alcohol consumption and all-cause mortality in 14 European countries. Addiction, 2001. 96(1s1): 113-128.
10. Norström, T. and M. Ramstedt, Mortality and population drinking: a review of the literature. Drug and Alcohol Review, 2005. 24(6): 537 - 547.
11. Skog, O.J., The risk function for liver cirrhosis from lifetime alcohol consumption. Journal of Studies on Alcohol, 1984. 45(3): 199-208.

© 2018 Jiang H et al. *JAMA Network Open*.
